# Effects of distinct Polycystic Ovary Syndrome phenotypes on bone health

**DOI:** 10.3389/fendo.2023.1163771

**Published:** 2023-05-12

**Authors:** Edouard G. Mills, Ali Abbara, Waljit S. Dhillo, Alexander N. Comninos

**Affiliations:** ^1^ Section of Endocrinology and Investigative Medicine, Imperial College London, London, United Kingdom; ^2^ Department of Endocrinology, Imperial College Healthcare NHS Trust, London, United Kingdom; ^3^ Endocrine Bone Unit, Imperial College Healthcare NHS Trust, London, United Kingdom

**Keywords:** PCOS, bone, fracture, BMD, hyperandrogenism, hyperinsulinaemia, obesity, vitamin D

## Abstract

Polycystic Ovary Syndrome (PCOS) is a highly prevalent and heterogenous endocrinopathy affecting 5-18% of women. Although its cardinal features include androgen excess, ovulatory dysfunction, and/or polycystic ovarian morphology, women often display related metabolic manifestations, including hyperinsulinaemia, insulin resistance, and obesity. Emerging data reveal that the hormonal alterations associated with PCOS also impact bone metabolism. However, inconsistent evidence exists as to whether PCOS is a bone-protective or bone-hindering disorder with an accumulating body of clinical data indicating that hyperandrogenism, hyperinsulinaemia, insulin resistance, and obesity may have a relative protective influence on bone, whereas chronic low-grade inflammation and vitamin D deficiency may adversely affect bone health. Herein, we provide a comprehensive assessment of the endocrine and metabolic manifestations associated with PCOS and their relative effects on bone metabolism. We focus principally on clinical studies in women investigating their contribution to the alterations in bone turnover markers, bone mineral density, and ultimately fracture risk in PCOS. A thorough understanding in this regard will indicate whether women with PCOS require enhanced surveillance of bone health in routine clinical practice.

## Introduction

Polycystic Ovary Syndrome (PCOS) is a heterogenous endocrinopathy primarily characterised by a combination of signs and symptoms of androgen excess and ovulatory dysfunction in the absence of other specific diagnoses (1). Depending on the diagnostic criteria employed, current estimates suggest that it affects 5-18% of women (2), making it one of the most prevalent endocrine conditions affecting women of reproductive age. As a heterogenous condition, the aetiology is complex and multifactorial, and includes genetic and epigenetic factors, neuroendocrine dysfunction, androgen excess, insulin resistance and obesity (2–4).

Several diagnostic criteria have been developed, with the most widely used being based on the European Society of Human Reproduction and Embryology and the American Society for Reproductive Medicine consensus workshop group from 2013, known as the Rotterdam Criteria (5). Diagnosis is confirmed with two of three criteria: (I) oligo- and/or anovulation, (II) clinical and/or biochemical signs of hyperandrogenism and (III) polycystic ovarian morphology (5). Based on the Rotterdam criteria, four main phenotypes exist: phenotype A (hyperandrogenism, ovulatory dysfunction, and polycystic ovarian morphology); B (hyperandrogenism and ovulatory dysfunction); C (hyperandrogenism and polycystic ovarian morphology); D (ovulatory dysfunction and polycystic ovarian morphology) (6). Furthermore, women with PCOS can be further categorised according to body mass index (BMI) as having either overweight/obese or lean body mass (7).

Considering the established roles that reproductive hormones such as kisspeptin, androgens, and oestrogens play in skeletal homeostasis (8–10), it is unsurprising that akin to other reproductive disorders, the hormonal alterations in PCOS can also influence bone metabolism. However, inconsistent evidence of its effect on bone mineral density (BMD) exists with previous studies suggesting either a negative effect (11–16), a positive effect (17), or even no effect (18–25). Similarly, studies investigating long-term fracture risk as a hard clinical endpoint are equally discrepant. In a large register-based and population-based cohort study of 76,682 Danish women (19,199 with PCOS and 57,483 age-matched controls), fracture rates were lower in women with PCOS (10.3 versus 13.6 per 1000 patient years) (26). Indeed, the adjusted odds ratio was 0.76 (95% confidence interval [CI], 0.71 to 0.80) for all fractures, 0.82 (95% CI, 0.74 to 0.92) for major osteoporotic fractures, and 0.57 (95% CI, 0.74 to 0.70) for head and face fractures (26). In stark contrast, in another nationwide population-based cohort analysis involving 55,530 Taiwanese Chinese women (11,106 with PCOS and 44,424 age-matched controls), the PCOS group were noted to exhibit an increased risk of fractures (27). Specifically, the adjusted hazard ratio was 1.23 (95% CI, 1.13 to 1.33) for any fractures, 1.33 (95% CI, 1.15 to 1.54) for osteoporotic fractures, 1.36 (95% CI, 1.11 to 1.66) for spinal fractures and 1.39 (95% CI, 1.07 to 1.80) for forearm fractures (27). It is therefore difficult to reconcile these incongruous results. However, it is striking that Chinese women with PCOS display lower rates of obesity, impaired glucose tolerance, and insulin resistance, as well as a milder hyperandrogenic phenotype than women with PCOS of other ethnicities (28–30), suggesting that a plethora of factors must influence bone health in this heterogenous disorder.

To this end, in this review we provide a comprehensive assessment of the endocrine and metabolic manifestations associated with PCOS and their relative effects on bone metabolism. We focus principally on human clinical studies investigating their contribution to the alterations in bone turnover markers, bone mineral density, and fracture risk in women with PCOS. A thorough understanding of these factors may permit the development of novel targeted therapeutic strategies to optimise bone health in women with PCOS.

## Methods

We performed a literature review and identified relevant publications by means of a PubMed search for English-language articles using the following search terms: (“polycystic ovary syndrome” OR “PCOS” OR “PCO”) AND (“bone” OR “BMD” OR “turnover” OR “fracture” OR “vitamin D”) AND (“testosterone” OR “hyperandrogenism” OR “inflammation” OR “oxidative stress” OR “obesity” OR “adiposity” OR “insulin resistance”). Relevant data were subsequently extracted from the identified publications, and secondary data sources identified therein. To ensure inclusion of the most up-to-date data, searches were performed up until 10^th^ February 2023.

## Hyperandrogenism

Hyperandrogenism (clinical and/or biochemical) is one of the hallmark features of PCOS (31), observed in around 75% of cases (32, 33). Given that androgens have well-established roles in bone physiology (8), this lends credence to hyperandrogenism offering a positive influence on bone health in PCOS, either directly by binding to androgen receptors (AR) on bone-related cells, or indirectly through peripheral aromatization to oestrogens (34). Specifically, human osteoblasts express AR (35) with androgens capable of directly stimulating both murine and human osteoblastic cell proliferation *in vitro* (36). In contrast, whilst AR expression has been detected in rodent osteoclasts *in vitro* (37) and *in vivo* (38), no expression has been identified in human osteoclasts (39, 40), suggesting that the effect of androgens on osteoclastogenesis and bone resorption is mediated largely *via* cells of the osteoblast lineage in humans (41).

In a rodent model of PCOS, female rats receiving testosterone within nine days of birth were observed to develop polycystic ovaries and exhibited 15% higher BMD and lower bone turnover compared with controls (42). Likewise, using a similar experimental paradigm, a recent study revealed that postnatal androgenisation of female rats (achieved using a single dose of testosterone at day 5 of life) resulted in a marked increase in trabecular bone of the distal femur as evidenced by a 34% higher voxel bone volume to total bone volume, 24% more trabeculae, and 24% lower trabecular separation compared with control rats (43). From a mechanistic perspective, analysis of gene expression for bone formation and resorption factors in the rat femurs revealed reductions of *Dickkopf-1 factor* (a negative regulator of osteoblast differentiation) and *Interleukin 1-b* (an activator of osteoblast differentiation) (43). These preclinical findings suggest that androgen excess could serve as a protective factor in terms of bone health in PCOS, with several human clinical studies examining the effect of hyperandrogenism on bone as discussed below.

### Bone mineral density

BMD is the core component of bone strength and therefore protection against fractures. The relative importance of ovulatory cycles and circulating levels of androgens and oestrogens has been evaluated in data from Italy obtained from 51 women with PCOS, 24 women with idiopathic hirsutism (i.e., normal oestrogen and androgen levels, and ovulation), 26 women with Hypothalamic Amenorrhoea (HA, i.e., low oestrogen and androgen levels, and anovulation), and 35 healthy controls (matched for BMI, but significantly older than the other groups) (18). As expected, lumbar spine and femoral neck BMD were significantly lower in the women with HA, when compared with the three other groups. In contrast, the PCOS and idiopathic hirsutism groups had lumbar spine and femoral neck BMD values which were equivalent to the healthy controls (18). Indeed, in a subgroup of PCOS women with amenorrhoea (< 4 menstrual cycles per year, although the duration of amenorrhoea was not defined in the study), the BMD values remained comparable with the healthy controls, even after controlling for BMI and height (18). Furthermore, in the women with PCOS, lumbar spine BMD was associated with circulating androstenedione levels, and femoral neck BMD was associated with circulating free testosterone and dehydroepiandrosterone sulfate (DHEA-S), even once correcting for BMI and height (18). Collectively, these findings suggest that even in the amenorrhoeic women with PCOS, androgen excess may preserve bone mass (independent of BMI) and protect against possible bone loss due to other PCOS-associated factors.

**Figure 1:**
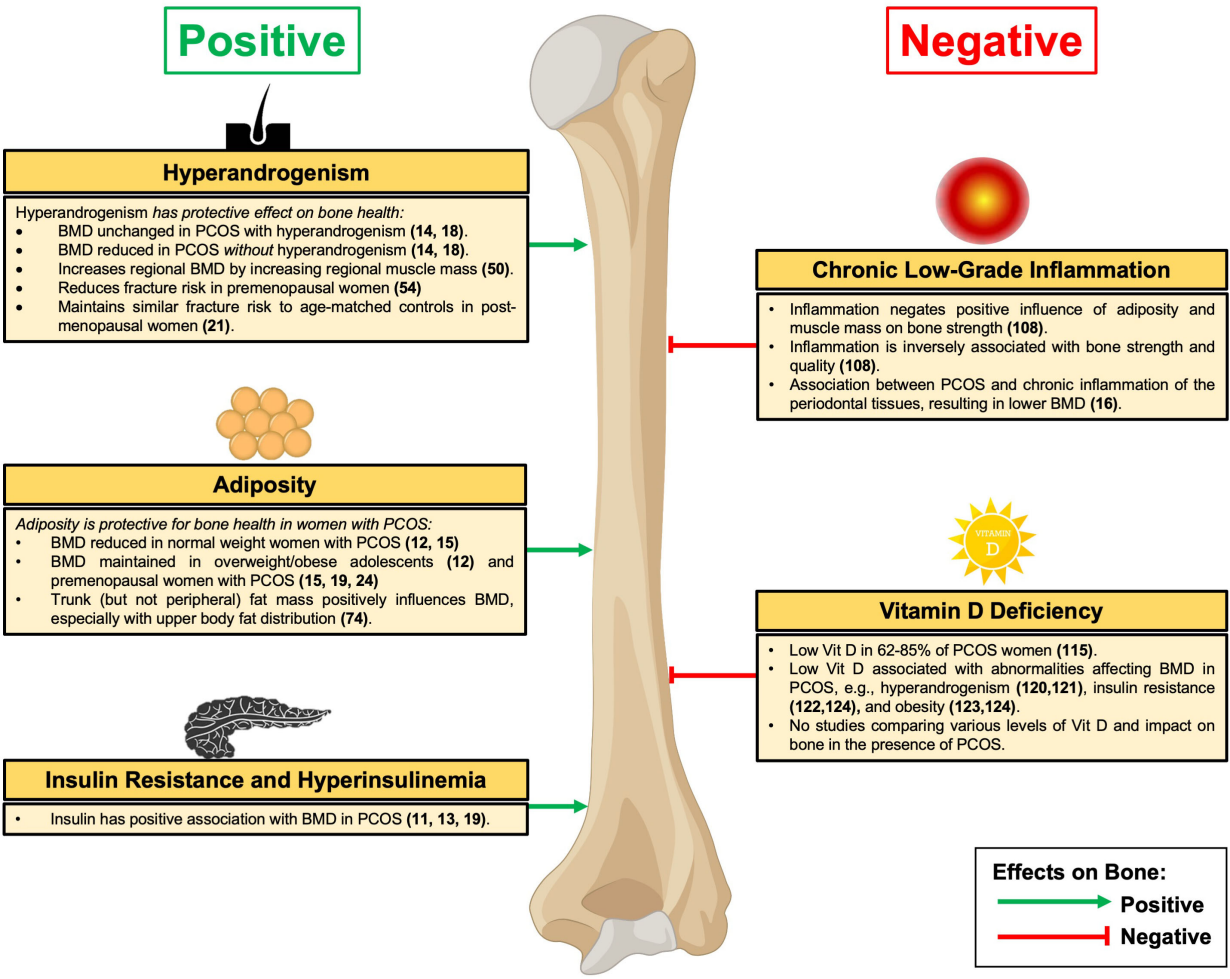
Effects of PCOS on Bone. BMD, bone mineral density; PCOS, Polycystic Ovary Syndrome. Green denotes relative positive effects on bone and red denotes relative negative effects on bone.

In a similar but larger cross-sectional study of 163 premenopausal women from Turkey (103 with PCOS and 60 age- and BMI-matched healthy controls), unlike the earlier mentioned study, lumbar spine and femoral neck BMD were 7.5% and 6% lower, respectively, in the PCOS group compared with controls (14). However, it is significant that in the subgroup of women with PCOS and hyperandrogenism, lumbar spine and femoral neck BMD were 9.5% and 11.3% higher, respectively, than in women with PCOS *without* hyperandrogenism [and comparable with the values observed in healthy controls, in keeping with the aforementioned study (18)]. Furthermore, in women with PCOS, there was a significant positive correlation between both lumbar spine and femoral neck BMD and serum oestradiol, total testosterone, androstenedione, and DHEA-S levels, as well as the Homeostatic Model Assessment for Insulin Resistance [HOMA-IR] (a robust surrogate method to estimate insulin resistance), providing evidence that insulin resistance acts as an important determinant of BMD in PCOS as discussed later in this review (14). Together, these data suggest that hyperandrogenism may protect against some of the negative effects on BMD associated with PCOS (summarised in **Figure 1**).

To eliminate the confounding effects of BMI and central obesity on BMD, further studies have been undertaken in the USA in lean women with PCOS, including 22 women (12 with PCOS and BMI < 26 kg/m^2^, and 10 healthy controls matched for age, ethnicity, and bodyweight) (17). Levels of total and bioavailable testosterone were 2.3- and 3.1-fold higher in women with PCOS, respectively, whereas DHEA-S, fasting glucose, and insulin levels, were comparable with controls (17). Interestingly, although there was no significant difference in total BMD, significantly greater upper skeletal BMD was noted in the PCOS group compared with controls, indicating that in lean women with PCOS, regional differences in BMD may exist with a protective effect of PCOS on upper body bone mass (17). This remains to be elucidated in future studies but again provides clinical evidence for a protective effect of elevated androgens on BMD.

In addition to bone-related cells, AR are expressed in numerous cell types within human skeletal muscle, including satellite cells, fibroblasts, CD34+ precursor cells, vascular endothelial, smooth muscle cells, and mast cells (44). This suggests that androgens increase muscle mass in part by targeting multiple cell types to modulate the differentiation of mesenchymal precursor cells in the skeletal muscle (44). Furthermore, in muscle, testosterone promotes muscle hypertrophy (45) through the combined effects of stimulating protein synthesis (i.e., anabolic effect) (46) and inhibiting protein degradation (i.e., anti-catabolic effect) (47). Indeed, it is well-established that muscle mass exerts independent effects on BMD through mechanical action (48, 49), with data from Japan examining the relationship of testosterone to regional muscle size and BMD in women with PCOS (50). In a study of 71 women with PCOS, serum testosterone levels (but not serum androstenedione or DHEA-S) were positively associated with lean (muscle) mass in each of the segmental regions examined (left arm, right arm, trunk, left leg, and right leg), as might be expected given testosterone’s established role in muscle mass. Moreover, regional lean (muscle) mass was observed to correlate significantly with BMD, even after adjusting for age, height, and weight (50). Collectively, this suggests that testosterone in addition to more direct bone effects can also positively influence regional BMD through increasing regional muscle mass in women with PCOS (summarised in **Figure 1**).

### Fracture risk

Fractures are the final consequence of bone fragility. Several studies have explored fracture risk (together with BMD) in PCOS. Although PCOS is considered a female disorder, it has been proposed that the upstream endocrine and metabolic disturbances can also arise in men (7, 51). Genetic studies reveal that first-degree relatives of women with PCOS, including men, carry increased risk of glucose intolerance, insulin resistance, cardiovascular disease, and male pattern baldness (52, 53). Using data from UK Biobank, a recent study of women and men employed a Mendelian randomisation approach to examine the association of genetic risk of hyperandrogenism in PCOS with BMD and fracture risk (54), with the single nucleotide polymorphisms identified being from the largest genome-wide association study for PCOS to date (55). This resulted in a study population of 221,086 Caucasian women and 187,816 Caucasian men, of whom 11% and 10% self-reported fractures over the last 5 years, respectively. Furthermore, a one standard deviation increase in genetic risk (adjusted for age and BMI) for hyperandrogenism in PCOS was associated with significantly higher BMD, and a significantly reduced risk of all-cause fractures (odds ratio 0.97, 95% CI, 0.96 to 0.99) in women. In contrast, excess genetic risk for hyperandrogenism in PCOS with higher BMD and reduced fracture risk was not observed in men (54), which may be because men inherently are exposed to greater androgens and so increased exposure may have little or no additional effect on bone. This study examined all-cause fractures, therefore additional studies using a Mendelian randomisation approach are warranted to establish whether the protective effect of hyperandrogenism in PCOS occurs in both traumatic and non-traumatic (fragility) fractures.

The vast majority of studies exploring the effects of PCOS on bone metabolism have been undertaken in premenopausal women. In a prospective 21-year follow-up study (performed initially in 1987 with the participants re-examined in 2008) from Sweden investigating whether 25 now post-menopausal women with PCOS (aged 61 to 78 years) differed from 68 aged-matched controls regarding BMD and fracture risk, at follow-up the free androgen index (FAI) remained 42% higher in the PCOS group (21). However, despite maintaining a markedly higher FAI, post-menopausal women with PCOS had similar muscle mass (body fat and lean mass), BMD, and fracture risk compared with controls. This may suggest that hyperandrogenism plays a less significant protective effect on bone and muscle mass during the oestrogen-deprived postmenopausal years [given that oestrogens up-regulate androgen receptors (56)], resulting in similar BMD and fracture incidence to age-matched controls (21).

## Adiposity

Evidence from the prospective population-based Northern Finland Birth Cohort 1996 revealed that weight gain in early adulthood plays a crucial role in the emergence of PCOS in later life (57). Consistent with this, epidemiological estimates suggest that 38-88% of women with PCOS have overweight or obesity (58, 59). Outside of PCOS, the relationship between obesity and bone metabolism is complex and depends on several factors, including mechanical loading and higher lean mass (which are associated with better outcomes), the obesity type (i.e., peripheral versus abdominal obesity) and low-grade systemic inflammation (60, 61). Moreover, in addition to osteoblasts, chondrocytes, myocytes, and fibroblasts, mesenchymal stem cells also differentiate into adipocytes. Adipocytes secrete numerous proinflammatory cytokines such as interleukin 1 beta (IL-1β) and tumour necrosis factor alpha (TNF-α), as well as adiponectin and leptin (62). Regarding effects on bone, adiponectin has been shown in mice to increase bone mass by suppressing osteoclastogenesis and by stimulating osteoblastogenesis (63), whereas leptin has positive (64, 65) or negative (66, 67) effects depending on the leptin status and the mode of action (central or peripheral) (68). To this end, a significant number of studies examining the effect of PCOS on bone have been undertaken in women with overweight or obesity and are summarised below.

### Bone metabolism

Fibroblast growth factor 23 (FGF23) is a bone-derived phosphaturic hormone predominantly secreted by osteocytes and osteoblasts (69), which has been shown to be related to abdominal adiposity in women with PCOS (70). In this age-matched analysis from Bulgaria of 40 women with PCOS without obesity, 20 women with PCOS and obesity (BMI > 30 kg/m^2^), and 20 control women with obesity alone, FGF23 levels (and serum calcium, parathyroid hormone [PTH], vitamin D, receptor activator of nuclear factor kappa-B ligand [RANKL] and osteopontin) were similar between the PCOS and non-PCOS groups (70). When grouping women with PCOS based on the presence of abdominal adiposity (waist circumference > 80 cm), FGF23 levels were 34% higher compared with the women with waist circumferences < 80 cm. Similarly, in women with PCOS at increased risk of cardiovascular disease, assessed according to the Androgen Excess and PCOS Society consensus (71), FGF23 was 40% higher and vitamin D 32% lower compared with women with PCOS not at increased cardiovascular disease risk (70). Beyond levels of bone-related blood markers, BMD was not assessed in this study, which would be a fruitful area for further investigation given the inconsistent clinical evidence regarding the effects of FGF23 on bone mass and fragility fractures in non-PCOS cohorts (the elderly and patients with chronic kidney disease) (72). Overall, whether FGF23 is an important putative factor in bone health in PCOS, especially in women with obesity where FGF23 levels are higher, remains to be determined.

### Bone mineral density

Several studies have investigated whether there are any differences in BMD between normal weight and women with PCOS and obesity. In a prospective cohort study from Hong Kong of 77 adolescents aged between 16 and 18 years (37 with newly diagnosed PCOS and 40 age-matched normal-weight healthy controls), lumbar spine BMD (but not femoral neck, distal radius, or distal tibia) was significantly lower in the PCOS group (12). However, in the weight-stratified analysis, whereas normal weight women with PCOS displayed lower BMD at the lumbar spine and trochanter as compared with the healthy controls, the overweight (using a BMI cut-off > 25 kg/m^2^) PCOS group had similar BMD as controls (12). Importantly, circulating total testosterone was 73% higher in the overweight PCOS group compared with the healthy controls (12), suggesting that hyperandrogenism and raised BMI may have a combined protective effect on bone. Of note, FAI or free testosterone was not presented in this study, although likely to be higher given the lower sex hormone binding globulin (SHBG) frequently observed in PCOS (73). Similarly, in a meta-analysis of 134,180 women (31,383 with PCOS and 102,797 controls), women with PCOS and BMI < 27 kg/m^2^ had lower total femoral and lumbar spine BMD values as compared with the controls, whereas comparatively in women with PCOS and BMI > 27 kg/m^2^ no difference with controls was observed (15). From a mechanistic perspective, osteocalcin (an osteoanabolic marker) levels were significantly reduced in women with PCOS and BMI < 27 kg/m^2^ (but not BMI > 27 kg/m^2^) compared with controls, suggesting that the reduced BMD in lower-weight PCOS may in part be due to reduced bone formation (15). Furthermore, in a study from India of 118 women (60 with PCOS and 58 age-matched controls), while no difference in BMD between the PCOS and control groups was observed, BMD values at the lumbar spine, femoral neck, and total hip, were significantly greater in PCOS women with BMI > 25 kg/m^2^ than in women with PCOS and BMI < 24.9 kg/m^2^ (24). Across the whole cohort of women with PCOS, lumbar spine, femoral neck, and total hip BMD correlated positively with BMI, waist circumference, and hip circumference (but not with fasting insulin, HOMA-IR, serum testosterone or DHEA-S levels) (24). Taken together, this series of studies suggests that BMI is an important determinant of BMD in women with PCOS with increasing adiposity playing a protective role by either increasing BMD or maintaining BMD to similar levels of non-PCOS healthy controls (summarised in **Figure 1**).

Furthermore, it is intriguing to consider the separate effects of trunk and peripheral fat mass on BMD in women with PCOS. In a study from Japan investigating the non-weight effects between trunk and peripheral mass on BMD (using the arm as a non-weight bearing site) in 123 women with PCOS, trunk fat mass was noted to positively correlate with arm BMD (74). Of note, this relationship was most marked in women with upper body fat distribution than in those women with low body fat distribution. In contrast, peripheral fat mass was unrelated to BMD in this study cohort, suggesting that in PCOS, trunk fat mass influences BMD more than peripheral fat mass, which may relate to differences in mechanical stresses on bone (74).

## Insulin resistance and hyperinsulinaemia

Insulin resistance and the resulting compensatory hyperinsulinaemia occurs in approximately 65% of women with PCOS (75). Compared with women with PCOS *without* insulin resistance, those with insulin resistance display greater β-cell function, which is compatible with their ability to maintain higher circulating insulin levels (75). Moreover, women with insulin resistance are also observed to be more obese with a greater android body fat distribution and are more androgenised (in terms of circulating androgens, hirsutism, and acne) (75). These findings are consistent with the established effect of the compensatory hyperinsulinaemia stimulating androgen secretion from ovarian thecal cells (76, 77), making insulin resistance and hyperinsulinaemia key drivers of excess androgens in PCOS (3). Regarding bone, insulin receptors are expressed on rodent osteoblasts (78, 79) and osteoclast-like cells (80), as well as human osteoblast precursors and mature osteoblasts (81). Consistent with this, insulin-signalling has anabolic effects and has been shown to promote osteoblast differentiation and osteocalcin expression in mouse calvarial osteoblasts *in vitro* (82). Moreover, mice lacking insulin receptors on osteoblasts have been shown to have low circulating undercarboxylated osteocalcin levels and reduced bone acquisition due to diminished bone formation with deficient osteoblasts numbers (79). Turning to humans, in patients with type 1 diabetes mellitus (i.e., characterised by insulin deficiency), BMD is decreased, whereas in type 2 diabetes (i.e., characterised by insulin resistance and so possibly akin to PCOS with regards to bone) BMD is often normal or elevated compared with age-matched controls (83). To this end, the clinical studies examining the influence of insulin resistance and hyperinsulinaemia in PCOS are summarised below.

### Bone metabolism

Osteocalcin is an osteoblast-derived protein which has modulatory roles in the control of bone turnover and supports the later stage of bone formation by acting at the bone mineral surface (84). More recently, osteocalcin has been implicated for its role in regulating insulin secretion and action (85). Consistent with this, clinical data from Greece highlight that serum concentrations of carboxylated osteocalcin (the predominant bone-active form) are increased and associated with several components of PCOS (86). In a study of 97 women (50 with PCOS and 47 age- and BMI- matched healthy controls), although circulating total osteocalcin was 44% lower in the PCOS group, carboxylated osteocalcin was 75% higher (unlike other measures of bone biochemistry such as PTH, osteoprotegerin, and vitamin D levels which were similar), independent of obesity (86). Moreover, significant associations were identified between carboxylated osteocalcin with fasting insulin levels, insulin resistance (determined by HOMA-IR), and circulating testosterone levels (86). Collectively, these findings identify a putative two-way interaction between bone-derived markers and the metabolic alterations observed in PCOS which warrants further study.

### Bone mineral density

Several investigators have examined the relationship between insulin resistance and BMD in PCOS, revealing a relative protective effect against bone loss. In a study from Turkey consisting of 54 premenopausal women (28 amenorrhoeic women with PCOS, 11 amenorrhoeic women without PCOS, and 15 healthy controls), lumbar spine and femoral neck BMD in the PCOS group were lower than in healthy controls, but higher than in amenorrhoeic women without PCOS (11). Notably, the three groups were similar in terms of age and BMI. Regarding insulin concentrations, mean fasting insulin levels were 48% higher in the PCOS group than in the healthy controls and 39% higher than in the amenorrhoeic controls, whereas the insulin sensitivity index (determined during an insulin tolerance test) was significantly lower. This is in keeping with the established insulin excess/resistance observed in PCOS. Furthermore, in the PCOS group there was a positive correlation between lumbar spine BMD with insulin and a negative correlation with insulin sensitivity index (11). Congruent with these findings, in a separate study from Poland of 99 premenopausal women (69 women with PCOS and 30 age-matched healthy controls), the lumbar spine BMD values were 16% lower in the PCOS group overall than in the controls (13). However, in a BMI-stratified analysis, only the women with PCOS and normal weight had a lower BMD than the controls, whereas BMD in the women with overweight and obesity was equivalent to the healthy controls (providing further evidence for the protective effect of adiposity). Similar to the earlier data from Turkey, a positive correlation between lumbar spine BMD and serum insulin levels and HOMA-IR was observed (13). Moreover, it is significant that even in those studies, where BMD measurements do not differ between women with PCOS and healthy controls, that the association between insulin resistance and hyperinsulinaemia with BMD persists. For instance, in an analysis of 46 women from Turkey (29 with PCOS and 17 age- and BMI-matched healthy controls), fasting insulin concentrations were 40% higher in the PCOS group and the fasting glucose/insulin ratio was significantly lower (19). Despite comparable BMD values (total, lumbar spine, and femoral neck), there were significant positive and negative correlations between fasting insulin and total BMD and fasting glucose/insulin ratio and lumbar spine BMD, respectively, after controlling for age and BMI (19), suggesting that insulin resistance and hyperinsulinaemia might play a role in the preserved BMD (summarised in **Figure 1**).

## Chronic low-grade inflammation

Chronic low-grade inflammation is increasingly recognised as a perpetuator of the endocrine and metabolic abnormalities observed in PCOS (87). For instance, women with PCOS display elevations in circulating C-reactive protein (CRP), which is 96% higher compared with controls, an effect which is independent of obesity (88). Furthermore, a relationship between altered bone metabolism and chronic inflammation has been observed in numerous widespread diseases, such as inflammatory bowel disease, multiple sclerosis, and rheumatoid arthritis (89, 90). From a molecular perspective, inflammation is associated with overproduction of cytokines, such as interleukin 1 beta (IL-1β), 6 (IL-6) and 17 (IL-17), and TNF-α, which are stimulators of osteoclastogenesis mainly by promoting the expression of RANKL and macrophage colony stimulating factors (M-CSF) (91–95). Similarly, inflammatory chemokines, including the CC class, are also involved in promoting osteoclastogenesis (96). In addition to these established effects on osteoclast activity, several cytokines including TNF-α have been shown to suppress osteoblast differentiation and trigger osteocyte apoptosis (95, 97, 98). Taken together, these inflammatory mediators have significant effects on bone remodelling by driving the system towards resorption (99). Accordingly, a number of studies have examined the effect of chronic low-grade inflammation on bone in the context of PCOS.

### Bone metabolism

Osteoprotegerin is an inhibitor of osteoclastic bone resorption with increasingly recognised anti-inflammatory roles (100, 101). In a case-control study from Spain of 80 women (40 with PCOS and 40 age- and BMI-matched healthy controls), serum osteoprotegerin levels were 16% lower in PCOS, an effect which was independent of obesity (102). Of note, serum levels of RANKL and RANKL:osteoprotegerin ratio were similar in both cohorts, suggesting that the reduced osteoprotegerin levels are independent of an increase in serum RANKL concentration and availability. In addition, multivariable linear regression of both groups revealed that PCOS, obesity, and age were significant determinants with 20.8% of the variation in serum osteoprotegerin levels explained by differences in these variables (102). Therefore, given the emerging anti-inflammatory effects of osteoprotegerin, the reduced serum levels seen in this study might contribute to the chronic low-grade inflammation seen in PCOS. To what extent the difference in serum osteoprotegerin levels in PCOS may influence or correlate with BMD remains to be determined in future studies, particularly given that higher circulating osteoprotegerin levels are often found in patients with osteoporosis (103).

Growth/Differentiation Factor-15 (GDF-15), also known as non-steroidal anti-inflammatory drug-inducible gene-1, is a member of the transforming growth factor β superfamily, which has diverse physiological functions in pregnancy, and in pathological conditions (including inflammation, myocardial ischemia, and cancer) (104, 105). As to its role in bone, GDF-15 has been shown to positively regulate osteoclast differentiation in osteocytic cells under hypoxia (106). In a study from Turkey of 42 women with PCOS and obesity, plasma levels of GDF-15 were shown to not differ significantly with 23 women with idiopathic hirsutism and 20 healthy controls (matched for age and BMI) (107). In addition, markers of bone formation (serum bone-specific alkaline phosphatase [ALP] and osteocalcin) and bone resorption (urine deoxypyridinoline and pyridinoline), as well as BMD (femoral neck, total hip, and lumbar spine) were similar in all three groups. However, it is notable that in the subgroup of women with PCOS, plasma GDF-15 levels were negatively correlated with circulating osteocalcin and positively correlated with urine deoxypyridinoline (107), in keeping with the evidence discussed earlier relating to the positive regulation of osteoclastic differentiation by GDF-15 in osteocytic cells (106). Collectively, these data identify GDF-15 as another putative marker in the crosstalk between bone and chronic inflammation in PCOS.

### Bone mineral density

To determine whether the aforementioned changes in bone metabolism compromises BMD, the effect of chronic low-grade inflammation has been investigated in 61 women (22 with PCOS and 39 controls) (108). In this study from Canada, the presence of inflammation as assessed by CRP and the CRP/albumin ratio were two-fold greater in the women with PCOS compared with controls, whereas there was no difference in markers of oxidative stress (leukocyte telomere length and urinary 8-hydroxydeoxyguanosine) (108). Furthermore, in a multivariable linear regression model of the entire study cohort, a diagnosis of PCOS negatively predicted hip BMD when weight was included (108). Indeed, in the women with PCOS, inflammation negated the positive influences of increased weight and muscle mass on bone strength and was inversely associated with the radial strength-strain index (a measure of bone strength and quality independent of body weight on bone size) (108). From a clinical perspective, abdominal adiposity, and concurrent oral oestrogen therapy [both of which are associated with increased CRP levels (109, 110)] were shown to be independent predictors of subclinical inflammation, suggesting the need to consider the metabolic manifestations of PCOS and also its treatments (discussed later in this review) on long-term bone health (108).

Chronic periodontitis is a host-inflammatory condition occurring when untreated localised inflammation of the gums progresses to loss of the gingiva, bone, and ligaments supporting the teeth, and can eventually result in tooth loss (111). A well-established association between chronic periodontitis and PCOS exists, which is relevant given that a proinflammatory environment is common to both conditions (112). Recent evidence from India provides the first data investigating the effect of PCOS on bone metabolism in the presence of chronic periodontitis (16). In this study, 40 women with PCOS and chronic periodontitis were compared with 40 women with PCOS alone, 40 women with chronic periodontitis alone, and 20 control women (16). Circulating levels of serum C-terminal telopeptide (CTx, bone resorption marker) were higher in the PCOS and chronic periodontitis group, whereas serum levels of bone-specific ALP were lower (16). BMD at the lumbar spine and femoral regions was significantly lower in women with PCOS and chronic periodontitis when compared with the other groups, indicating that chronic inflammation in PCOS negatively effects bone (16), possibly through bone resorption owing to its ability to induce osteoclast differentiation with increased serum CTx as discussed earlier (summarised in **Figure 1**).

## Vitamin D deficiency

Vitamin D plays a critical role in calcium-phosphate homeostasis and optimal bone health by facilitating intestinal calcium absorption, reducing urinary calcium losses, and mobilising skeletal calcium stores (113). In contrast, vitamin D deficiency is associated with low bone density and fracture risk as a result of secondary hyperparathyroidism and high bone turnover with impaired bone mineralisation (114). From a clinical perspective, estimates highlight that 62-85% of women with PCOS have a serum 25-hydroxy vitamin D level < 20 ng/ml (115) and so considered vitamin D deficient (116). Furthermore, genetic studies have established an association between polymorphisms of the vitamin D receptor (*VDR*) gene and PCOS (117). For instance, the genetic variant *rs757343* has been observed to be associated with the severity of the PCOS phenotype (but not an increased risk for PCOS) (118), while variants in the *Cdx2* and *DHCR7* genes are associated with insulin resistance and insulin sensitivity in PCOS (119).

As discussed throughout this review, the interplay between PCOS and bone metabolism is an emerging field of study, however, no studies have been undertaken to determine the effects of PCOS on bone specifically in the presence of vitamin D deficiency. Despite this, there is an association between vitamin D and several endocrine and metabolic abnormalities that impact bone health in PCOS. Regarding hyperandrogenism, vitamin D is positively associated with SHBG and negatively with the FAI in women with PCOS (120). Accordingly, in a recent randomised placebo-controlled clinical study, 12 weeks of vitamin D supplementation (50,000 IU per week) was observed to significantly lower hirsutism scores and androgen levels of overweight women with PCOS and vitamin D deficiency (121). Turning to insulin resistance, meta-analytical data demonstrate that women with PCOS and vitamin D deficiency are more likely to have dysglycaemia (including elevated fasting glucose and HOMA-IR) compared with women with PCOS without vitamin D deficiency (122). Likewise, in a cross-sectional analysis of women with PCOS, vitamin D levels were significantly lower in women with PCOS and obesity or insulin resistance than in counterparts without obesity or insulin resistance (123). In an additional meta-analysis of 10 randomised controlled trials comparing the effects of vitamin D supplementation versus placebo, both low dose (< 4000 IU/day) and high dose (> 4000 IU/day) vitamin D were found to significantly reduce fasting glucose and total cholesterol levels (but not BMI, blood pressure, fasting insulin, triglycerides, or HOMA-IR) (124). Taken together, given the prevalence of vitamin D deficiency in women with PCOS, it is possible that low vitamin D levels can directly affect bone health in this patient cohort, or indirectly through its relationship with hyperandrogenism, insulin resistance, and obesity, as discussed above (summarised in **Figure 1**).

## Age at diagnosis

PCOS can be diagnosed at any age from menarche onwards, with recent estimates of the global disease burden across 194 countries highlighting that the highest incidence occurs among women aged 15-19 years (125). The age at diagnosis is therefore important given that a change in health status during attainment of peak bone mass may be associated with lifelong lower bone density and increased fracture risk (126). Data from the US National Health and Nutrition Examination Survey indicates that the age at attainment of peak femoral neck, total hip and lumbar BMD are 18.7, 19.0 and 20.1 years in females, respectively (127). Along these lines, a small collection of studies has investigated whether young age influences bone health in women with PCOS as described below.

### Bone metabolism

In a multicentre study from Finland and Sweden of 492 women (298 with PCOS and 194 healthy controls), serum levels of the bone formation markers procollagen type 1 N propeptide (P1NP) and osteocalcin were 19% and 13% lower in the PCOS group, respectively, even after adjustment for age and BMI (128). By comparison, levels of the bone resorption marker CTx were equivalent (128). Of note, age-stratified analyses demonstrated that the differences in P1NP and osteocalcin were largely due to the differences observed in the younger age group (< 30 years), even after adjusting for BMI (128). Specifically, serum P1NP and osteocalcin levels were 22% and 16% lower in the women aged < 30 years compared with controls, respectively, whereas no differences were observed in other age groups (31-40 years and 40-menopause). Interestingly, after adjusting for BMI, P1NP and osteocalcin were shown not to correlate with testosterone, DHEA-S or FAI, indicating that circulation androgens may not be associated with the decreased bone formation markers observed in younger women with PCOS (128).

### Bone mineral density

Given the data indicating decreases in bone formation markers (but equivalent resorption markers) in younger women, it is interesting to consider whether this translates into lower BMD as one might expect. In an analysis of four cross-sectional studies from the USA consisting of 170 females aged 12 to 25 years (123 with PCOS and 47 controls), total BMD and BMD z-scores were similar in both groups (25). As expected, HOMA-IR and androgens (free and total testosterone) were significantly raised in the PCOS group (25). Interestingly, simple, and multiple regression analyses were fitted to identify putative predictive factors associated with BMD z-scores, which revealed that obesity status and insulin resistance were each independent predictors, whereas PCOS status or free testosterone levels did not independently affect the BMD z-scores (25). In fact, it was demonstrated that for each unit increase in HOMA-IR, the BMD z-score increased by 0.06 and that having obesity increased the BMD z-score by 0.41 (25). Taken together, this suggests that in adolescents and young women, PCOS may disrupt bone metabolism but not enough to lower BMD, whereas obesity and insulin resistance may instead have beneficial effects (25).

### Fracture risk

In a large population-based study from Denmark, which included 76,682 women (19,199 with PCOS and 57,483 age-matched controls), fracture risk was observed to be lower in women with PCOS compared with controls (10.3 versus 13.6 per 1000 patient-years), especially in the appendicular skeleton (26). From an age perspective, the fracture risk reduction was most pronounced in those women with a younger age at diagnosis (adjusted hazard ratio for any fracture < 30 years 0.66 [95% CI, 0.60 to 0.71] versus 0.86 [95% CI, 0.76 to 0.93] in the women aged > 30). Remarkably, the risk reduction was not confined to traditional BMD-dependent fracture sites (forearm, lower leg, femur, and hip), but also traditionally BMD-independent fracture sites (hands, head, and face). Collectively, this implies that the relative protective effects associated with PCOS (such as secondary to hyperinsulinaemia, hyperandrogenism, and adiposity) may be more prominent in women who are yet to reach or have just reached peak bone mass (26).

## Effects of PCOS treatments on bone

### Weight loss

Lifestyle modification and weight loss are often regarded as the first line treatment in PCOS. However, as discussed earlier, given that adiposity is positively associated with BMD in PCOS, studies have been undertaken to determine the effect of weight loss on bone, particularly given that weight loss can acutely decrease bone mass in non-PCOS adults (129). In a recent study from Canada examining 60 premenopausal women with PCOS who were randomised to either a pulse-based diet (e.g., chickpeas, split peas, and lentils) or therapeutic lifestyle changes diet for 16-weeks while following an aerobic exercise programme, both diets resulted in 5% weight reduction at follow-up (130). This was associated with statistically significant small improvements in lumbar spine BMD and bone mineral content with both interventions, but also a statistically significant loss in femoral neck BMD (1% in the therapeutic lifestyle changes diet group and 2% in the pulse-based diet group) (130). Given that pulse-based diets are associated with lower fasting insulin (131), this could suggest that the combined effect of reducing both weight and hyperinsulinaemia can have a detrimental effect on bone. However, longer duration studies are required to investigate this further. To this end, a similar study from Sweden examined the effects of a 12-month structured weight loss programme with a very low energy diet in 246 women with obesity (63 with PCOS and 183 without PCOS) (132). In the 72 women (16 with PCOS and 56 without PCOS) who completed the study at follow-up, the weight reduction was 11% in the PCOS group and 13% in the non-PCOS group (132). Interestingly, in the women without PCOS, significant reductions in circulating androgens (total and free testosterone and FAI), fasting insulin, and HOMA-IR, were observed after 12-months of interventions, along with a diminished total bone mass (132). In contrast, circulating androgens, fasting insulin, and HOMA-IR, were not significantly different in the PCOS group and the total bone mass was preserved (132). Taken together, this may indicate that the elevated androgens and hyperinsulinaemia in women with PCOS may have a protective effect on bone mass in the context of certain weight loss programmes.

### Vitamin D supplementation

There is growing evidence that the benefits of vitamin D supplementation on BMD is only observed in the context of vitamin D deficiency (133, 134). Therefore, given that women with PCOS manifest a high prevalence of vitamin D deficiency, which likely influences bone *via* direct mechanisms or indirectly through its relationship with hyperandrogenism, insulin resistance, and obesity as discussed earlier, ensuring vitamin D sufficiency (and supplementing accordingly) may optimise bone health in PCOS. Importantly, randomised clinical trials with large sample sizes of women with PCOS and vitamin D deficiency should seek to explore the dose-response effects of vitamin D supplementation on bone turnover markers, bone mineral density, and long-term fracture risk.

### Metformin

Metformin is a biguanide medication that is commonly prescribed in PCOS to increase insulin sensitivity by decreasing gluconeogenesis, lipogenesis, and enhancing hepatic glucose uptake (135, 136). Along these lines, the effects of metformin on serum concentrations of bone turnover markers have been investigated in 118 premenopausal women with PCOS (74 without obesity and 44 with obesity) as part of a multicentre study from Finland whereby the women were randomised to receive either metformin or placebo for 3-months (137). Of note, after 3-months of metformin treatment, levels of P1NP and CTx were significantly decreased in both those with and without obesity, whereas no significant differences were seen in the placebo group (137). Specifically, the decline from baseline values were 27% for P1NP and 30% for CTx, an effect which was shown to be independent of whether the women had normal or high androgen levels (137). Collectively, these findings indicate that metformin results in reduced overall bone turnover as suggested by reduced levels of bone formation as well as resorption markers (137). Therefore, given that low bone turnover is associated with a slower rate of bone loss, the addition of metformin to lifestyle modification (caloric restriction and exercise) over 6 months has been investigated in 114 premenopausal women with PCOS (55 metformin and 59 placebo group) (138). In this study from the USA, metformin resulted in improvements in insulin sensitivity as expected, but without any significant benefit on circulating androgen levels. From a bone perspective, a small (but significant) increase in total BMD was observed in the metformin group (whereas a small decrease was seen in the placebo group) (138), in keeping with the aforementioned study of bone turnover markers indicating that metformin is unlikely to have a detrimental effect on bone but may have a small benefit (based on these short duration studies).

### Steroidal contraceptives

In PCOS, treatment with steroidal contraceptives is used to regularise menstrual cycles and lower free testosterone by increasing SHBG, which in theory may influence BMD (139). In a cross-sectional analysis from Australia of 95 premenopausal women with PCOS and overweight or obesity who either recently took steroidal contraceptives (stopped 3 months prior) or were not taking steroidal contraceptives, total BMD was 8% lower for women with recent steroidal contraceptives (140). Furthermore, lower BMD was demonstrated to be independently associated with contraceptive uses, lower BMI, and higher testosterone (but not age, multivitamin, calcium intake or vitamin D, HOMA-IR, calcium, and alcohol) (140). These findings are surprising given that steroidal contraceptives are frequently used to promote BMD maintenance (in sex steroid deficient states) and hyperandrogenism is regarded as having a protective effect on bone in PCOS. However, it is important to note that high doses of sex steroids in steroidal contraceptives can suppress upstream reproductive hormones and other related osteoanabolic factors such as kisspeptin and IGF1 (8, 9). Moreover, the contraceptive formulation in this study varied (in terms of androgenic, progestin and oestrogen) with each likely to have different effects on bone (141, 142). Finally, total BMD (as assessed in the aforementioned study) is regarded as a less sensitive marker of bone health than BMD at specific measurement sites (e.g., lumbar spine and femoral neck) (140). Therefore, the clinical significance of these findings should be examined further in randomised controlled trials with assessment of bone turnover markers and site-specific BMD at baseline and following steroidal contraceptive use versus placebo.

### Pioglitazone

Pioglitazone is a peroxisome proliferator-activated receptor-γ agonist (PPARγ) that can be used as an insulin sensitising treatment in PCOS (143). Evidence from animal models reveals that PPARγ agonist treatment results in impaired osteoblast function but unaltered osteoclast activity (144, 145). Osteoprotegerin is an inhibitor of osteoclastic bone resorption with increasingly recognised anti-inflammatory roles (100, 101). Given that pioglitazone reduces the inflammatory state (143), studies have evaluated circulating osteoprotegerin levels before and after treatment with pioglitazone 30mg or placebo for 16-weeks (146). In this analysis from Denmark of 44 premenopausal women (30 with PCOS and 14 age- and BMI-matched healthy controls), plasma osteoprotegerin levels were comparable between groups and were unaltered by pioglitazone treatment (146). However, it is significant that in a separate analysis by the same group, pioglitazone treatment in these patients resulted in a 7% reduction in total ALP and 14% reduction in PTH (but had no effect on osteocalcin levels), suggesting a possible small decrease in osteoblast activity (147) in keeping with animal models above. Regarding BMD, significant decreases were observed in the lumbar spine and femoral neck (1.1%, 1.4%, respectively), compared with placebo, despite unchanged testosterone and oestradiol levels (147). Taken together, given that pioglitazone appears to result in adverse effects on bone health by altering bone balance resulting in bone loss has led to the recommendation that dual-energy X-ray absorptiometry assessment should be taken at baseline followed by 2-yearly intervals if PPARγ agonist treatment is instigated in PCOS (143). Therefore, from a bone-perspective, metformin would be the preferred insulin-sensitising agent.

## Conclusions and future directions

PCOS is a highly prevalent and heterogenous endocrine disorder amongst women of reproductive age. Although its cardinal features include androgen excess, ovulatory dysfunction, and/or polycystic ovarian morphology, women with PCOS often exhibit metabolic manifestations, such as hyperinsulinaemia, insulin resistance, and obesity. As discussed in this review, an accumulating body of evidence indicates that hyperandrogenism, hyperinsulinaemia, insulin resistance, and obesity appear to have a beneficial impact on bone, whereas chronic low-grade inflammation and vitamin D deficiency may adversely affect bone (summarised in **Figure 1**). In this review, we have discussed the relative contributions of each of these factors based on the available evidence. Clearly separating the factors from each other is difficult given their frequent coexistence in PCOS and so we have highlighted the many caveats to the studies reviewed. As shown in **Table 1**, together with the heterogeneity of PCOS in terms of phenotype, it is therefore unsurprising to find studies suggesting that overall PCOS can have a negative effect, a positive effect, or no effect, on BMD with similar inconsistent evidence regarding long-term fracture risk when PCOS is taken as a whole. This review has therefore served to highlight the distinct contributions of each factor on bone to gain some clarity on the effects.

**Table 1:** PCOS phenotypes and subtypes and related effects on bone.

PCOS Phenotype	Associated Characteristics	Relative Effects on Bone
NIH’s Evidence-based Methodology Workshop on PCOS (5)		
**A**	Hyperandrogenism Ovulatory dysfunction Polycystic ovarian morphology	*Hyperandrogenism:* • Promotes trabecular bone growth and lowers bone turnover in rodent models (42, 43). • Maintains BMD to similar levels of healthy controls (unlike in PCOS women *without* hyperandrogenism where BMD is reduced) (14, 18). • Positively influences regional BMD through increasing regional muscle mass (50). • Significantly reduces the risk of all-cause fractures in premenopausal women (54) or maintains a similar BMD and fracture risk to age-matched controls in post-menopausal women (21). *Ovulatory dysfunction:* • PCOS women with amenorrhoea have similar BMD (independent of BMI) to healthy controls (unlike women with HA where BMD is reduced) (18), suggesting that the deleterious effect on bone of amenorrhoea is balanced by other PCOS-related factors (e.g., androgens, insulin, and obesity). *Polycystic ovarian morphology:* • Not known to have direct effects on bone.
**B**	Hyperandrogenism Ovulatory dysfunction	*Hyperandrogenism:* • Promotes trabecular bone growth and lowers bone turnover in rodent models (42, 43). • Maintains BMD to similar levels of healthy controls (unlike in PCOS women without hyperandrogenism where BMD is reduced) (14, 18). • Positively influences regional BMD through increasing regional muscle mass (50). • Significantly reduces the risk of all-cause fractures in premenopausal women (54) or maintains a similar BMD and fracture risk to age-matched controls in post-menopausal women (21). *Ovulatory dysfunction:* • PCOS women with amenorrhoea have similar BMD (independent of BMI) to healthy controls (unlike women with HA where BMD is reduced) (18), suggesting that the deleterious effect on bone of amenorrhoea is balanced by other PCOS-related factors (e.g., androgens, insulin, and obesity).
**C**	Hyperandrogenism Polycystic ovarian morphology	*Hyperandrogenism:* • Promotes trabecular bone growth and lowers bone turnover in rodent models (42, 43). • Maintains BMD to similar levels of healthy controls (unlike in PCOS women without hyperandrogenism where BMD is reduced) (14, 18). • Positively influences regional BMD through increasing regional muscle mass (50). • Significantly reduces the risk of all-cause fractures in premenopausal women (54) or maintains a similar BMD and fracture risk to age-matched controls in post-menopausal women (21). *Polycystic ovarian morphology:* • Not known to have direct effects on bone.
**D**	Ovulatory dysfunction Polycystic ovarian morphology	*Ovulatory dysfunction:* • PCOS women with amenorrhoea have similar BMD (independent of BMI) to healthy controls (unlike women with HA where BMD is reduced) (18), suggesting that the deleterious effect on bone of amenorrhoea is balanced by other PCOS-related factors (e.g., androgens, insulin, and obesity). *Polycystic ovarian morphology:* • Not known to have direct effects on bone. *Absence of hyperandrogenism*: • BMD significantly lower in PCOS women *without* hyperandrogenism than PCOS women *with* hyperandrogenism (14).
Overweight/Obese and Lean Subtypes (7)		
**Overweight/Obese**	++ insulin resistance and hyperinsulinaemia ++ chronic low-grade inflammation	*Adiposity:* • BMD comparable with healthy controls in both overweight/obese adolescents (12) and premenopausal adults (15, 19, 24) with PCOS (unlike normal weight PCOS women where BMD is lower). • Trunk (but not peripheral) fat mass positively influences BMD, especially in women with upper body fat distribution (74). *Insulin resistance and hyperinsulinaemia* • Positive relationship between fasting insulin and insulin resistance with BMD in PCOS (11, 13, 19). *Chronic low-grade inflammation* • Presence of inflammation negates the positive influence of increased adiposity and muscle mass on bone strength and is inversely associated with bone strength and quality (108). • An established association between PCOS and the pro-inflammatory condition chronic periodontitis exists and results in significantly lower BMD than compared with PCOS *without* chronic periodontitis or chronic periodontitis *without* PCOS (16).
**Lean**	+ chronic low-grade inflammation + insulin resistance and hyperinsulinaemia	*Lean:* • Lower lumbar spine, femoral and hip BMD in lean PCOS women compared with overweight/obese PCOS women (15, 24). • Higher upper skeletal BMD in lean PCOS women compared with weight-matched healthy controls (17). *Insulin resistance and hyperinsulinaemia* • Positive relationship between fasting insulin and insulin resistance with BMD in PCOS (11, 13, 19). *Chronic low-grade inflammation* • Presence of inflammation negates the positive influence of increased adiposity and muscle mass on bone strength and is inversely associated with bone strength and quality (108). • An established association between PCOS and the pro-inflammatory condition chronic periodontitis exists and results in significantly lower BMD than compared with PCOS *without* chronic periodontitis or chronic periodontitis *without* PCOS (16).

BMD, bone mineral density; BMI, body mass index; HA, Hypothalamic Amenorrhoea; NIH, National Institutes of Health; PCOS, Polycystic Ovary Syndrome; ++ indicates prominent features and + indicates features that are present but less prominent.

Of note, vitamin D deficiency is present with all phenotypes with the effects on bone health displayed in **Figure 1**.

Furthermore, some of these discordant findings may also be due to differences in the diagnostic criteria for PCOS employed, the skeletal sites examined, and various degrees of hormonal imbalance and medication use in the women studied. As mentioned above, PCOS is a heterogenous disorder in terms of phenotypes, clinical manifestations, and metabolic consequences. Hence, the cumulative effect of PCOS on bone health is difficult to clearly define. Furthermore, much of the data is derived from cross-sectional observational studies (which are associated with a multitude of confounders), meaning that only associations of interest can be investigated, rather than causative mechanisms. Further studies utilising a Mendelian randomisation approach to test the association of genetic risk for specific risk factors could be a way of isolating specific risk factors and overcoming the confounding factors associated with observational studies. Moreover, much of our understanding relating to PCOS and bone metabolism comes from studies conducted in Asia, Europe, and North America, and given the genetic and ethnic differences associated with PCOS, additional studies in other diverse geographical locations are warranted.

To this end, there is conflicting data on the effect of PCOS on BMD and fracture risk. Owing to phenotypic heterogeneity of PCOS, future studies in the field should investigate specific PCOS phenotypes in isolation to clarify the various impacts on bone to aid individual patient management. These data will be key to defining to what extent women with PCOS require surveillance of bone health and bone-directed medications in routine clinical practice.

## Author contributions

EM researched the material and wrote the first draft. AC supervised all aspects of the work and is the corresponding and senior author. All authors contributed to the article and approved the submitted version.
